# Pancreatic β‐cell Function is Higher in Morning Versus Intermediate Chronotypes With Obesity

**DOI:** 10.1002/osp4.70064

**Published:** 2025-02-26

**Authors:** Steven K. Malin, Mary‐Margaret E. Remchak, Emily M. Heiston, Chiara Fabris, Ankit M. Shah

**Affiliations:** ^1^ Department of Kinesiology & Health Rutgers University New Brunswick New Jersey USA; ^2^ Division of Endocrinology, Metabolism & Nutrition Rutgers University New Brunswick New Jersey USA; ^3^ New Jersey Institute for Food Nutrition and Health Rutgers University New Brunswick New Jersey USA; ^4^ Institute of Translational Medicine and Science Rutgers University New Brunswick New Jersey USA; ^5^ Center for Diabetes Technology School of Medicine University of Virginia Charlottesville Virginia USA

**Keywords:** glycemic control, incretins, insulin resistance, insulin secretion, type 2 diabetes

## Abstract

**Objectives:**

People with later chronotypes are at greater T2D risk, yet it is unknown if β‐cell function differs among chronotypes. Thus we, assessed β‐cell function in morning (MORN) and intermediate (INT) chronotypes with obesity.

**Methods:**

Adults (*n* = 41, 9M, 55 ± 1.7 y, 36.8 ± 1.0 kg/m^2^) were grouped as MORN or INT per the Morningness‐Eveningness Questionnaire. Glucose, insulin, C‐peptide, GIP, and GLP‐1(active) were collected every 30 min during a 120 min 75g‐OGTT. Insulin secretion rates (ISR) were calculated (regularized deconvolution) to assess early (total area under the curve; tAUC_0–30min_) and total‐phase (tAUC_0–120min_) glucose‐stimulated insulin secretion (GSIS:ISR/Glucose). Skeletal muscle (glucose infusion rate/steady‐state insulin) insulin sensitivity and hepatic (HOMA‐IR) as well as adipose (Adipose‐IR) insulin resistance were assessed during a 120 min euglycemic hyperinsulinemic clamp (40mU/m^2^/min, 90 mg/dL). β‐cell function (disposition index (DI): GSIS adjusted insulin sensitivity) was determined. Body composition (DXA) and fitness (VO_2_max) were also measured.

**Results:**

Age, body composition and VO_2_max were similar between groups, but INT had reduced muscle insulin sensitivity and higher hepatic and adipose IR (*p* < 0.05). INT had higher C‐peptide tAUC_0–30min_ (*p* = 0.04) and lower hepatic DI (tAUC_0–30min_ *p* = 0.05 and tAUC_0–120min_ *p* = 0.07, respectively). Early phase hepatic DI correlated with GLP‐1 tAUC_0–30min_ (*r* = 0.35, *p* < 0.02) and tAUC_0–120min_ (*r* = −0.40, *p* = 0.04).

**Conclusions:**

β‐cell function was higher in MORN versus INT chronotypes. Further work is warranted to discern how chronotype impacts insulin secretion.

**Trial Registration:**

NCT03355469

## 1 Introduction

Chronotype reflects the behavioral and physiologic processes that influence preference to engage in lifestyle behaviors across the day. Specifically, those with evening chronotypes (i.e. “night owls”) prefer to wake up and/or engage in activity later in the day compared with those with morning chronotypes (i.e. “early bird”) who engage in most acts of living during the morning hours. People with intermediate chronotypes tend to engage in activities during both early and later portions of the day. Evening chronotypes have a higher risk of type 2 diabetes than morning or intermediate chronotypes due to, in part, insulin resistance [1, 2]. Interestingly, the central clock (e.g. suprachiasmatic nucleus) regulates whole‐body insulin sensitivity through in part neural input, while peripheral clocks in muscle, adipose and liver determine tissue specific insulin action [3]. However, improving β‐cell function to secrete insulin relative to low skeletal muscle, liver and/or adipose insulin sensitivity is critical to promote glycemic control [4]. In this regard, focus on pancreatic function warrants attention for the regulation of insulin secretion since misalignment between daily rhythms of sleep, eating, or movement may raise the risk of type 2 diabetes.

Glucose tolerance is typically higher in the morning than in the evening among healthy people [5, 6], and this time of day dependent effect is primarily driven by declines in pancreatic β‐cell responses to nutrients [7, 8, 9]. Rodent [10] and human pancreatic islet studies [11] demonstrate that the transcription factors CLOCK and BMAL1 activate gene expression that regulates insulin biosynthesis, transport and glucose‐stimulated insulin secretion [12]. Despite work suggesting pancreatic β‐cells are under circadian system regulation and people with obesity/type 2 diabetes have blunted diurnal patterns [8, 9, 13, 14], no work has been done to examine glucose‐stimulated insulin secretion and pancreatic function in people with different chronotypes. This limits insight into how the β‐cell responds to glucose via the triggering pathway for insulin secretion as well as if incretin hormones that act on amplifying pathways of the β‐cell, such as GLP‐1 (glucagon‐like polypeptide‐1) and/or GIP (glucose insulinotropic polypeptide), differ among chronotypes [15]. Previously, it was shown using the euglycemic‐clamp and ultrasound approaches that people with early chronotypes have higher metabolic insulin sensitivity compared with people with later chronotypes, and those with morning chronotype have more favorable insulin‐stimulated endothelial function than those with intermediate chronotypes [2, 16]. Subsequently, it would be reasonable to anticipate that people with intermediate chronotypes (INT) would have lower pancreatic β‐cell function compared with morning (MORN) chronotypes. This is important because people with obesity have reduced early and late phase insulin secretion, thereby raising the risk of type 2 diabetes [17]. Thus, attention to INT has public health significance as nearly 50% of the population identifies within this classification [18]. The purpose of this study was to determine if people with MORN and INT would differ in early and total phase β‐cell function. It was hypothesized that people with the MORN chronotype would have different β‐cell functions than those with INT chronotype, and this would relate to the incretins GLP‐1 and GIP.

## 2 Methods

### 2.1 Participants

Sedentary individuals were enrolled in this cross‐sectional study. People with obesity were recruited by flyers, social media, and/or electronic medical records from the local communities. To classify participants as either MORN or INT, we used the Morningness‐Eveningness Questionnaire (MEQ) as previously described [2] from participants with available plasma for C‐peptides (Table 1). The Epworth Sleepiness Scale was used to assess the likelihood of falling asleep during specific daily events (i.e. watching tv, sitting inactive, in a car while stopped, etc.), and the Pittsburgh Sleep Quality Index (PSQI) was used to assess quality and patterns of sleep [19, 20]. Participants underwent a physical exam prior to exercise testing that included an overnight 12 h fasted blood/urine chemistries (e.g. liver enzymes (alkaline phosphatase (ALP), aspartate aminotransferase (AST), alanine transaminase (ALT)), HbA1c, total triglycerides, total cholesterol, VLDL cholesterol, LDL cholesterol, and HDL cholesterol) and resting electrocardiogram (ECG). Blood pressure was also determined. Participants were excluded from the study if they were physically active (exercise > 1 h/wk) [21], smoked, weight unstable (> 2 kg weight change during last 3 months), diagnosed with type 2 diabetes, or currently taking medications known to influence insulin sensitivity or endothelial function (e.g., biguanides, calcium channel blockers, α‐blockers, β‐blockers, etc.). Female participants were asked to indicate menses status (e.g. menopause; Table 1). Women were not on oral contraceptives, nor did any report use of hormone replacement treatment. Participants provided both written and verbal informed consent as approved by the Institutional Review Board. This study was part of a larger clinical trial (Registration # NCT03355469).

**TABLE 1:** Morning and intermediate chronotype characteristics.

	MORN	INT	*p*‐value	Cohen's *d* effect size
Participant demographics				
N (F)	25 (21F)	16 (11F)		
Age (yrs)	52.4 ± 1.2	53.3 ± 1.8	0.68	0.13
Menopausal status (%)				
Pre	44	50		
Peri	12	12		
Post	44	38		
Race/Ethnicity				
African American	2	2		
Hispanic/Latino	1	1		
White	22	13		
MEQ score	63.6 ± 0.9	51.7 ± 1.1	< 0.001	1.59
Epworth sleep score	6.7 ± 0.7	6.7 ± 1.0	0.95	0.01
PSQI	5.7 ± 0.5	7.5 ± 1.2	0.22	0.48
3‐Factor sleep efficiency	1.3 ± 0.3	1.5 ± 0.4	0.66	0.15
3‐Factor sleep quality	2.2 ± 0.5	3.2 ± 0.6	0.26	0.39
3‐Factor daily disturbances	2.2 ± 0.2	2.6 ± 0.2	0.20	0.42
Body Mass and composition				
Weight (kg)	101.8 ± 3.4	105.2 ± 5.2	0.58	0.18
BMI (kg/m^2^)	36.4 ± 1.0	36.7 ± 1.3	0.85	0.06
WC (cm)	111.2 ± 2.1	115.6 ± 3.6	0.30	0.36
Body fat (%)	43.9 ± 1.1	44.0 ± 1.1	0.92	0.03
Fat Mass (kg)	42.8 ± 1.9	44.2 ± 2.6	0.67	0.15
VAT (cm^2^)	1161.5 ± 122.4	1025.3 ± 88.2	0.37	0.27
Sub‐Q (kg)	2.8 ± 0.1	3.4 ± 0.4	0.19	0.54
LBM (kg)	52.3 ± 1.9	55.1 ± 3.6	0.51	0.25
Fitness, blood pressure, and clinical labs				
VO_2_max (mL/kg/min)	23.4 ± 0.8	22.2 ± 0.8	0.34	0.29
SBP (mmHg)	130.1 ± 2.8	135.8 ± 2.5	0.13	0.44
DBP (mmHg)	77.8 ± 1.9	81.1 ± 2.4	0.30	0.33
HbA1c (%)	5.7 ± 0.1	5.5 ± 0.1	0.45	0.29
TG (mg/dL)	152.1 ± 15.4	170.0 ± 16.9	0.45	0.24
Total cholesterol (mg/dL)	207.8 ± 6.7	225.3 ± 12.0	0.44	0.43
HDL (mg/dL)	45.6 ± 1.8	48.0 ± 2.3	0.40	0.26
TG:HDL ratio	3.5 ± 0.4	3.7 ± 0.4	0.77	0.09
LDL (mg/dL)	135.9 ± 5.0	148.7 ± 10.5	0.28	0.38
AST (U/L)	22.9 ± 1.3	28.0 ± 2.1	0.05	0.67
ALT (U/L)	28.1 ± 3.7	35.8 ± 3.3	0.13	0.45
ALP (U/L)	68.5 ± 4.1	68.5 ± 4.1	0.19	0.39

*Note:* Data are expressed as mean ± SEM.

Abbreviations: ALP = alkaline phosphatase; ALT = alanine aminotransferase; AST = aspartate aminotransferase; BMI = body mass index; DBP = diastolic blood pressure; HbA1c = hemoglobin A1c; HDL = high density lipoprotein; INT = intermediate chronotype; LBM = lean body mass; LDL = low density lipoprotein; MEQ = morning eveningness questionnaire; MORN = morning chronotype; PSQI = Pittsburgh sleep quality index; SBP = systolic blood pressure; SubQ = subcutaneous fat; TG = triglycerides; VAT = visceral fat; VO_2_max = maximal oxygen consumption.

### 2.2 Cardiorespiratory Fitness

Maximal oxygen consumption (VO_2_max) was used to determine cardiorespiratory fitness and was tested on a treadmill with indirect calorimetry. Individuals were asked to self‐select a speed and the incline was elevated every 2 min until exhaustion as described before [22].

### 2.3 Body Composition

A digital scale was used to assess body weight to the nearest 0.10 kg while participants wore minimal clothing and no shoes. Height was assessed to the nearest 0.10 cm using a stadiometer to ultimately calculate body mass index (BMI). Total body fat and lean body mass (LBM) were measured using dual‐energy X‐ray absorptiometry (Horizon DXA System, Marlborough, MA). Waist circumference (WC) was defined 2 cm above the umbilicus. WC was obtained twice and averaged using a tape measure to the nearest 0.10 cm.

### 2.4 Metabolic Control

A diet consisting of 55% carbohydrates, 15% protein, and 30% fat, with < 10% from saturated fat, was provided to standardize food consumption 24 h prior to the clamp and oral glucose tolerance test (OGTT). Food intake need was based on fasted resting metabolic rate (RMR) derived from indirect calorimetry, which was multiplied by a physical activity factor of 1.2. Individuals were also asked to avoid medication, caffeine, alcohol and engagement in moderate to vigorous physical activity 24 h prior to the study visits.

### 2.5 Euglycemic Clamp

After an overnight fast of about 10 h, participants arrived at the Clinical Research Center (CRC) as described previously [23]. A catheter was placed in the antecubital/forearm and dorsal hand veins for infusion and blood drawing, respectively. A primed (250 mU/m^2^/min) constant infusion (40 mU/m^2^/min) of insulin was delivered via peristaltic infusion pumps for 120 min. Plasma glucose samples were assessed every 5 min to maintain plasma glucose at 90 mg/d by adjusting the glucose infusion rate (GIR). Plasma insulin and free fatty acids (FFA) levels were also obtained at 0, 90, 105, and 120 min of the clamp, and values during the last 30 min were averaged to assess steady state levels. GIR was divided by steady‐state insulin to depict skeletal muscle (or peripheral) insulin sensitivity. Hepatic and adipose insulin resistance were estimated as we have done before by the product of fasting glucose and FFA by fasting insulin, respectively [2, 24, 25].

### 2.6 Pancreatic Insulin Secretion

On a separate day from the clamp, participants reported to our CRC after an overnight fast of about 10 h. An intravenous line was placed in the antecubital vein for blood collection in people in a semi‐supine position. Blood was obtained for the determination of plasma glucose, insulin, C‐peptide, GLP‐1(active) and GIP at 0, 30, 60, 90, and 120 min. Glucose‐stimulated insulin secretion (GSIS) was calculated using total area under the curve (tAUC) during the OGTT by way of the trapezoidal rule [2, 26]. C‐peptides were reconstructed by regularized deconvolution to depict insulin secretion rates (ISR) [27]. GSIS was defined by dividing ISR tAUC by glucose tAUC. GSIS during early (0–30 min) and total (0–120 min) phases were adjusted for skeletal muscle insulin sensitivity from the clamp as well as the liver and adipose tissue to collectively characterize skeletal muscle, hepatic and adipose pancreatic function. Adjusting GSIS to individual indexes of insulin sensitivity was conducted to understand the complex interplay between key glucose regulatory organs as done before [24, 25, 26]. Hepatic insulin clearance was estimated by dividing tAUC of C‐peptide by insulin during the OGTT.

### 2.7 Biochemical Analysis

Plasma glucose was analyzed by a glucose oxidase assay (YSI Instruments 2700, Yellow Springs, OH). The remaining samples were centrifuged at 4°C for 10 min at 3000 RPM and stored at −80°C until later batched‐analyzed in duplicate to minimize variance within conditions. EDTA vacutainers with aprotinin were used to collect insulin, C‐peptide, and FFA. EDTA vacutainers with aprotinin and dipeptidyl peptidase‐4 (DPP‐IV) inhibitor were used for assessing GLP‐1(active, i.e. 7–36 and 7‐37 amide) and GIP. These hormones were measured using an ELISA (Millipore, Billerica, MA), while FFAs were assessed by a colorimetric assay (Fuji, Richmond, VA).

### 2.8 Statistical Analysis

R (The R Foundation, Vienna, Austria 2013) was used to analyze the data collected in the present work. Data were assessed for normality, and skewed data were log transformed for analysis. Independent, two‐tailed *t*‐tests were used to compare groups. Cohen's *d* effect size was calculated to assess physiological relevance between groups, and interpreted as small *d* = 0.2, medium *d* = 0.5, or large *d* = 0.8, respectively. Associations were determined using Pearson's correlation. Significance was set at *p* ≤ 0.05 and trends were accepted as 0.05 < *X* ≤ 0.10. Data were expressed as mean ± SEM.

## 3 Results

### 3.1 Participant Characteristics

People with MORN and INT chronotypes did not significantly differ for age, body mass or composition, VO_2_max, blood pressure, or lipid levels (Table 1). No group differences were noted for PSQI total composite score, sleep efficiency, or daytime drowsiness. However, INT had higher AST levels (*p* = 0.05, *d* = 0.67) and modest elevations in ALT (*p* = 0.13, *d* = 0.45) despite no difference in ALP levels.

### 3.2 Clamp‐Derived Insulin Sensitivity

Fasting or steady state plasma glucose or FFA values during the clamp between MORN and INT were similar. However, fasting insulin was elevated in INT versus MORN (*p* = 0.01). Skeletal muscle insulin sensitivity was also higher in MORN than INT (*p* = 0.05, Table 2). Moreover, hepatic (*p* = 0.01) and adipose insulin resistance (*p* = 0.03) were similarly lower in MORN versus INT (Table 2).

**TABLE 2:** Insulin sensitivity measures between morning and intermediate chronotypes.

	MORN	INT	*p*‐value	Cohen's *d* effect size
Glucose				
Fasted (mg/dL)	105.4 ± 2.9	105.6 ± 3.2	0.96	0.01
Steady state (mg/dL)	88.8 ± 0.7	88.1 ± 1.0	0.60	0.17
Insulin				
Fasted (uU/mL)	12.1 ± 1.0	23.0 ± 3.7	0.01	0.95
Steady state (uU/mL)	80.5 ± 3.8	87.6 ± 4.5	0.33	0.38
Free fatty acids				
Fasted (mEq/L)	0.67 ± 0.04	0.67 ± 0.05	0.98	0.005
Steady state (mEq/L)	0.16 ± 0.01	0.16 ± 0.01	0.78	0.10
Clamp‐derived insulin sensitivity				
GIR (mg/kg/min)	2.4 ± 0.2	2.0 ± 0.2	0.17	0.48
GIR/Insulin (mg/kg/min/uU/mL)	0.035 ± 0.003	0.024 ± 0.003	0.05	0.67
HOMA‐IR (a.u.)	3.2 ± 0.3	6.1 ± 0.9	0.01	0.95
Adipose‐IR (a.u.)	8.4 ± 1.3	13.4 ± 1.8	0.03	0.83

*Note:* Data are expressed as mean ± SEM. Euglycemic clamp was used to measure skeletal muscle insulin sensitivity. Homeostatic model of insulin resistance (HOMA‐IR) was calculated as fasting glucose *x* fasting insulin then divided by 405 to depict hepatic insulin resistance. Adipose‐IR was calculated as fasting FFA *x* fasting insulin to determine adipose insulin resistance.

Abbreviations: INT = intermediate chronotype; MORN = morning chronotype.

### 3.3 OGTT Derived Glucose, Insulin and C ‐Peptides

Consistent with HbA1c (Table 1), fasting glucose, 2‐h glucose or early and total phase glucose tolerance were comparable in MORN and INT (Table 3). However, consistent with clamp measures, insulin tended to be elevated in the fasted (*p* = 0.11, *d* = 0.59) and early phase insulin states (*p* = 0.11, *d* = 0.67) in INT compared with MORN during the OGTT. Fasting C‐peptides (*p* = 0.15, *d* = 0.48) along with an early phase C‐peptides (*p* = 0.04, *d* = 0.74) in INT paralleled circulating insulin versus MORN.

**TABLE 3:** Glucose metabolism during an OGTT between morning and intermediate chronotypes.

	MORN	INT	*p*‐value	Cohen's *d* effect size
Plasma glucose				
Fasted (mg/dL)	106.8 ± 3.2	100.9 ± 3.0	0.19	0.39
2‐h (mg/dL)	144.8 ± 7.5	125.5 ± 7.1	0.07	0.54
Early phase tAUC_0–30min_	4039.9 ± 137.9	4082.5 ± 170.7	0.84	0.06
Total phase tAUC_0–120min_	18878.0 ± 832.0	1741.9 ± 944.5	0.37	0.28
Plasma insulin				
Fasted (uU/mL)	13.3 ± 1.2	19.3 ± 3.4	0.11	0.59
2‐h (uU/mL)	88.1 ± 9.3	86.3 ± 17.0	0.93	0.03
Early phase tAUC_0–30min_	1409.5 ± 189.3	2554.4 ± 606.0	0.08	0.67
Total phase tAUC_0–120min_	9776.1 ± 815.0	13880.2 ± 2542.7	0.14	0.58
Plasma C‐peptide				
Fasted (ng/mL)	2.4 ± 0.1	2.8 ± 0.2	0.15	0.48
2‐h (ng/mL)	8.5 ± 0.5	8.7 ± 1.3	0.89	0.05
Early phase tAUC_0–30min_	122.0 ± 6.7	164.0 ± 18.9	0.04	0.74
Total phase tAUC_0–120min_	843.2 ± 41.3	936.6 ± 90.9	0.36	0.33
Insulin Secretion Rate				
Fasted (ng/mL/min)	0.2 ± 0.0	0.4 ± 0.0	0.03	0.81
2‐h (ng/mL/min)	0.4 ± 0.0	0.4 ± 0.1	0.93	0.03
Early phase tAUC_0–30min_	12.6 ± 0.7	16.9 ± 2.1	0.07	0.66
Total phase tAUC_0–120min_	66.3 ± 3.3	70.8 ± 7.5	0.58	0.20
GSIS				
Early phase tAUC_0–30min_	0.0031 ± 0.0001	0.0042 ± 0.0005	0.10	0.62
Total phase tAUC_0–120min_	0.0035 ± 0.0001	0.0041 ± 0.0005	0.31	0.38
Plasma GLP‐1				
Fasted (pg/mL)	8.7 ± 1.8	7.3 ± 1.4	0.55	0.19
2‐h (pg/mL)	7.6 ± 1.6	10.2 ± 2.4	0.39	0.30
Early phase tAUC_0–30min_	234.9 ± 26.2	292.2 ± 46.4	0.29	0.40
Total phase tAUC_0–120min_	946.8 ± 142.7	1271.7 ± 249.8	0.27	0.44
Plasma GIP				
Fasted (pg/mL)	77.8 ± 10.6	92.7 ± 12.8	0.37	0.28
2‐h (pg/mL)	334.8 ± 25.9	304.5 ± 34.2	0.48	0.23
Early phase tAUC_0–30min_	6928.4 ± 410.6	6814.0 ± 618.7	0.87	0.05
Total phase tAUC_0–120min_	36919.4 ± 2953.4	40719.6 ± 2352.8	0.32	0.32
Hepatic insulin clearance				
Early phase tAUC_0–30min_	10.7 ± 4.0	9.8 ± 0.9	0.56	0.21
Total phase tAUC_0–120min_	14.8 ± 2.0	11.6 ± 0.7	0.28	0.40

*Note:* Data are expressed as mean ± SEM.

Abbreviations: GIP = glucose insulinotropic polypeptide; GLP‐1 = glucagon‐like polypeptide; GSIS = glucose‐stimulated insulin secretion rate (tAUC of ISR divided by Glucose); INT = intermediate chronotype; ISR = insulin secretion rate derived from deconvolution of plasma C‐peptide; MORN = morning chronotype; tAUC = total area under the curve.

### 3.4 Pancreatic β‐cell Function

**FIGURE 1:**
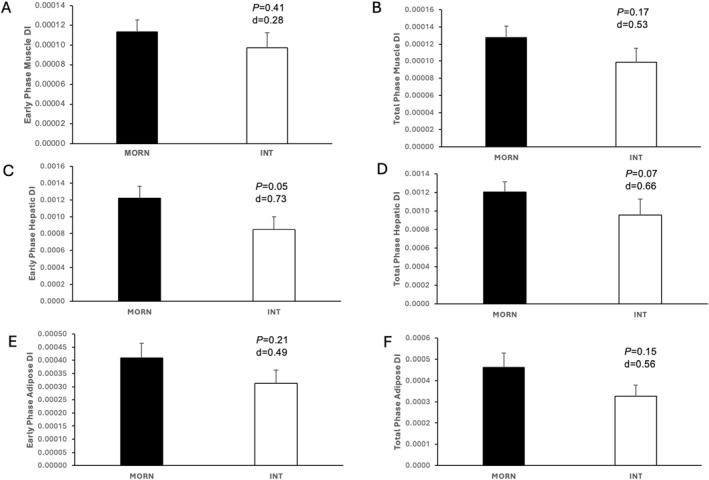
Morning versus Intermediate Chronotype on β‐cell function adjusted for multi‐organ insulin resistance. Data are expressed as mean ± SEM. DI = disposition index and was used to characterize pancreatic β‐cell function. Skeletal muscle DI was calculated as the clamp‐derived glucose infusion rate divided by steady‐state insulin and multiplied by glucose‐stimulated insulin secretion (GSIS) during the first 30 min and total 120 min of an oral glucose tolerance test. Hepatic DI was estimated as GSIS divided by HOMA‐IR. Adipose DI was determined as GSIS divided by Adipose‐IR. GSIS was calculated as tAUC of ISR/Glucose at the respective timepoints. All β‐cell function data were log‐transformed for statistical analysis, but are shown here in raw values.

In line with insulin and C‐peptides, fasting ISR was also elevated in INT versus MORN (*p* = 0.03, *d* = 0.81) as was early phase ISR (*p* = 0.07, *d* = 0.66; Table 3). Early phase GSIS mirrored higher levels in INT compared with MORN (*p* = 0.10, *d* = 0.62; Table 3), although there was no statistical difference in total phase GSIS. Early phase skeletal muscle and adipose disposition index were similar between MORN and INT, although there were medium effect sizes for both total phase disposition indexes (Figure 1). In contrast, the hepatic disposition index was significantly lower in INT compared with MORN (Figure 1). No difference in GLP‐1(active) or GIP was noted between MORN and INT, nor was hepatic insulin clearance.

### 3.5 Correlational Analysis

Early phase hepatic disposition index, but not skeletal muscle or adipose disposition index (data not shown), was related to early (*r* = 0.32, *p* = 0.05) and total tAUC GLP‐1 (*r* = 0.37, *p* = 0.03). AST was associated with higher early (*r* = 0.41, *p* = 0.01) and total phase insulin tAUC (*r* = 0.41, *p* = 0.01) as well as skeletal muscle insulin sensitivity (*r* = −0.36, *p* = 0.03), hepatic insulin resistance (*r* = 0.37, *p* = 0.02) and total phase skeletal muscle disposition index (*r* = −0.37, *p* = 0.02). ALT related to total phase insulin tAUC (*r* = 0.34, *p* = 0.02) and skeletal muscle insulin sensitivity (*r* = −0.39, *p* = 0.01) as well as hepatic insulin resistance (*r* = 0.31, *p* = 0.05) and adipose insulin resistance (*r* = 0.36, *p* = 0.05). ALT also related to skeletal muscle total phase disposition index (*r* = −0.38, *p* = 0.01). Skeletal muscle insulin sensitivity (*r* = −0.36, *p* = 0.02) as well as total phase skeletal muscle disposition index (*r* = −0.31, *p* = 0.05) correlated with circulating TG. The TG to HDL ratio was linked to fasting (*r* = 0.30, *p* = 0.05) and early phase ISR (*r* = 0.31, *p* = 0.04), total phase GSIS (*r* = 0.30, *p* = 0.05) and early (*r* = −0.34, *p* = 0.03) and total phase (*r* = −0.41, *p* = 0.01) skeletal muscle disposition index.

## 4 Discussion

The present work suggests that people with MORN chronotypes have lower early phase C‐peptides and higher β‐cell function adjusted for hepatic insulin resistance, independent of age, body composition and fitness when compared with individuals with INT chronotypes. Moreover, people with the MORN chronotype had higher skeletal muscle insulin sensitivity and lower hepatic and adipose insulin resistance. Despite lower C‐peptides and higher β‐cell function in those with MORN versus INT chronotype, glucose‐stimulated plasma GLP‐1(active) or GIP were not statistically different. However, plasma GLP‐1 tAUC did relate to β‐cell function when adjusted for hepatic insulin resistance. This is physiologically relevant as GSIS adjusted for multi‐organ insulin sensitivity provides an integrative view of glucose disposal in the body [2, 26]. Intriguingly, it has been reported using 24‐h hyperglycemic clamp approaches that glucose infusion rate, but not glucose disposal, changed over the day in people with type 2 diabetes [28], suggesting that the liver is a primary organ under circadian regulation. Thus, the higher pancreatic β‐cell function observed in those with MORN chronotype is clinically important since prior work shows that declines in insulin secretion capacity are a stronger determinant of type 2 diabetes than insulin sensitivity [29].

There were no reported differences in subjective assessments of sleep or doziness in this study, suggesting that sleep does not likely explain differences in insulin secretion among chronotypes. This is consistent with prior work reporting higher type 2 diabetes risk in people with later chronotypes independent of sleep [30]. Prior studies, however, examining protocols mimicking large shifts in behavioral and environmental circadian cycles (e.g. shift work) demonstrated that early phase post‐prandial insulin is lower in the circadian evening by nearly 27% compared with morning [31, 32]. Moreover, sleep restriction with circadian misalignment reduces insulin secretion capacity in healthy individuals, whereas circadian misalignment reduced insulin sensitivity only [33]. Melatonin is known to mediate sleep‐wake cycles, and it has been observed that lower nocturnal melatonin is related to insulin resistance [34] as well as people with later chronotypes [34]. This may be relevant to the current work since higher melatonin levels are reported in individuals with later versus early chronotypes during morning hours up to 12:00 [35]. Moreover, melatonin is suggested to decrease insulin secretion in cultured human islets [36], which is partly dependent on the gene variant melatonin receptor gene MTNR1B [36]. Interestingly, people with obesity and/or type 2 diabetes have been described to have inverted circadian systems, such that they are more insulin resistant and have lower insulin secretion in the morning than the evening (which is opposite of healthy controls) [13, 14, 28]. Taken together, additional work in people across chronotypes is needed to understand the physiology of circadian hormones related to glucose metabolism.

The current findings suggest that MORN chronotypes had higher pancreatic abilities to release the available pool of insulin granules (i.e. early phase) when compared with INT chronotypes, as evident by medium to large effect sizes. However, there appeared to be no difference between groups in the ability to synthesize insulin in response to ambient glucose during the OGTT (i.e. total phase). Considering the time‐course of insulin secretion may help explain why we observed differences in β‐cell function adjusted for hepatic insulin resistance compared with no overall difference in pancreatic function corrected for skeletal muscle or adipose insulin action. Indeed, adipose tissue requires subtle elevations above that of fasting insulin levels to suppress lipolysis [37], while normal late phase insulin secretion as depicted by our total phase indices should compensate for prevailing declines in skeletal muscle insulin sensitivity [38]. However, the reduced early phase insulin secretion in those with INT chronotype is of interest since the liver receives glucose initially upon absorption and requires ∼30–60 min after rises in insulin and glucose to be fully suppressed [38]. A possible factor limiting the early phase insulin secretion response could relate to the incretins GLP‐1 and GIP, which stimulate nearly 60% of post‐prandial insulin secretion [39]. In fact, GLP‐1 correlated with hepatic disposition index, suggesting that higher GLP‐1 levels may have contributed to people with MORN chronotype showcasing higher GSIS adjusted to hepatic insulin resistance. How chronobiology impacted incretins is beyond the scope of this work, but incretins do act on insulin secretion via amplifying pathways (e.g. cAMP, glutamate, etc.) compared with ingested glucose acting on triggering pathways (i.e. Ca^++^ mediated) [40]. Therefore, additional work examining the mechanism(s) by which chronobiology regulates pancreatic β‐cell function is warranted.

Another consideration is that the enzymes AST and ALT have conventionally been used to reflect hepatic injury and/or fatty infiltration [41], which has been linked to elevated FFA from visceral adipose tissue draining into the liver and inducing insulin resistance [42]. Interestingly, individuals with INT chronotype herein presented with higher AST levels and modest effects for ALT (albeit not‐statistically significant) when compared with people with MORN chronotypes. Although those with INT had similar visceral fat levels as determined from DXA when compared with people with the MORN chronotype, people with INT chronotype had higher adipose insulin resistance than those with the MORN chronotype. In fact, the similar FFA levels between chronotypes suggest that the excess insulin was needed, and sufficient to suppress lipolysis and/or clear FFA for re‐esterification among those with INT chronotype. However, it should be acknowledged that AST is not only found in liver tissue but also in other tissues such as skeletal muscle, gut, heart, bone, brain and β‐cells [43]. Indeed, AST is considered an important enzyme in pancreatic β‐cells that plays roles in transferring amino acids to the formation of citrate levels, which contributes to insulin secretion [39]. Prior work demonstrates that people with later chronotypes had elevations in glutamine as well as the TCA intermediates: isocitrate, alpha‐ketoglutarate, succinate, fumarate, malate and oxaloacetate [2]. These later observations of metabolites along with higher AST in INT herein support the idea that people with INT chronotype have alterations in β‐cell metabolism [15]. Indeed, elevations in AST and ALT have been associated with type 2 diabetes risk as well as declines in insulin secretion as estimated from fasting glucose and insulin (i.e. HOMA‐B) [44].

There are limitations to this study. The groups comprised mostly women of different menstrual status. However, the percentage of pre‐, peri‐ and post‐menopausal status was similar, suggesting that menstrual status is unlikely to be a main factor contributing to group differences. This study also did not have a lean control reference group to identify the role of body fat. Therefore, these findings may not be extrapolated to adults with varying weight status. Moreover, this study did not include people with evening chronotype, and people did present with impaired fasting glucose. It is generally viewed that individuals with evening chronotypes have a higher risk for type 2 diabetes than people with INT or MORN chronotypes, and the modest elevations in blood glucose suggest impairments in β‐cell function are not unanticipated. However, it remains important to note that despite no overall differences in glucose tolerance or HbA1c, people with INT chronotype demonstrated declines in both insulin sensitivity and pancreatic function more so than those with MORN. Testing was conducted in the morning hours, and it is possible that physiologic responses to a glucose load between chronotypes may differ if testing was conducted in the afternoon. An OGTT was used in this study to assess pancreatic function, and these results may not be generalizable to a mixed‐meal despite generally comparable responses as discussed before [45]. Furthermore, using intravenous glucose methods would enable the assessment of insulin secretion independent of gut hormones. However, the OGTT provides ecological value and is physiologically relevant for assessment of incretins. Lastly, HOMA‐IR and Adipose‐IR approaches [2, 26] may under‐ or over‐estimate differences in organ‐specific insulin action compared with stable isotopes despite being valid assessments.

In conclusion, people with MORN chronotypes have lower fasting insulin secretion rates and higher pancreatic β‐cell function when adjusted for hepatic but not skeletal muscle or adipose insulin sensitivity independent of age, body composition, fitness, or sleep. Interestingly, GLP‐1 related to higher pancreatic β‐cell function and lower AST correlated with lower GSIS. Together, these data suggest that people with MORN chronotype present with higher pancreatic function to regulate glycemia among people with obesity. Additional work is needed to clarify the role of chronotype on the crosstalk between pancreatic β‐cells and insulin sensitive tissues in order to optimize treatments that lower type 2 diabetes risk in people with obesity.

## Author Contributions

Conceptualization (S.K.M.); methodology (S.K.M.); formal analysis, (M.M.E.R., C.F., and S.K.M.); investigation (M.M.E.R., E.M.H., A.M.S. and S.K.M.); writing–original draft preparation (M.M.E.R. and S.K.M.); writing–review and editing (E.M.H., C.F., and A.M.S.); supervision (S.K.M.); project administration (S.K.M.); funding acquisition (S.K.M.). All authors have read and agreed to the published version of the manuscript.

## Conflicts of Interest

The authors declare no conflicts of interest.

## Data Availability

These data have not been made publicly available. However, the corresponding author (SKM) can provide further information on the data upon reasonable request.
